# Understanding individual-level absorptive capacity: unpacking the roles of innovative attitude and motivation in perceived academic performance of Chinese international students in South Korea

**DOI:** 10.3389/fpsyg.2026.1687241

**Published:** 2026-02-18

**Authors:** Mingyeong Jeon, Meiling Yin

**Affiliations:** Business School, Sejong University, Seoul, Republic of Korea

**Keywords:** individual-level absorptive capacity, innovative attitude, moderated mediation, motivation, perceived academic performance, PLS-SEM

## Abstract

As higher education internationalizes, the number of Chinese students in Korea continues to grow; however, limited research has examined how their absorptive capacity influences perceived academic performance. This study integrates insights from knowledge management and psychology literature to investigate how Chinese students’ absorptive capacity affects perceived academic performance. Survey data from 202 Chinese students enrolled in Korean universities were analyzed using PLS-SEM, revealing that absorptive capacity positively influences perceived academic performance (*β* = 0.287, *p* < 0.001), with innovative attitude partially mediating this relationship (*β* = 0.204, *p* < 0.001). The moderated mediation analysis indicates that motivation (*β* = 0.081, *p* < 0.05) further amplifies the link between absorptive capacity and perceived academic performance through innovative attitude. These findings suggest that external capabilities alone are insufficient to foster innovation and academic performance; instead, students’ internal learning capacity and motivation are crucial drivers of academic success.

## Introduction

International student mobility has become a defining feature of globalization, particularly in East Asian higher education systems such as South Korea (Statista, 2025), which have actively expanded international enrolment as part of national internationalization strategies. Existing research has largely conceptualized internationally mobile individuals as carriers of knowledge who contribute to the innovation capacity of host organizations, regions, and economies (Song et al., 2003; Oettl and Agrawal, 2008; Tzabbar, 2009; Storz et al., 2015). However, when international mobility is examined in educational settings, academic success cannot be assumed to follow automatically from exposure to new knowledge environments. Instead, students’ ability to acquire, assimilate, and transform unfamiliar academic knowledge, conceptualized as absorptive capacity, becomes central to effective learning (Zahra and George, 2002; Müller et al., 2021; Cavallo et al., 2025). Despite the rapid growth of international students in Korean universities, particularly from China, limited research has systematically examined how international students’ absorptive capacity operates at the individual level to shape academic performance in cross-border higher education contexts.

Prior research has predominantly conceptualized absorptive capacity at the organizational level, emphasizing its role in transforming external knowledge into innovative outcomes (Lo and Tian, 2020; Müller et al., 2021; Cavallo et al., 2025). Only a limited number of studies have extended the concept to the individual level to explain learning outcomes in educational settings, primarily from experiential learning and instructional design perspectives (Fernández-Mesa et al., 2022; Kim and Park, 2023). However, this emerging literature has largely treated absorptive capacity as a cognitive learning capability, leaving unclear how its effects are achieved through psychological mechanisms and under what conditions it shapes performance, particularly when learners are embedded in international educational environments.

Addressing this gap, the present study examines the relationship between individual-level absorptive capacity and perceived academic performance by integrating perspectives from education, management, and psychology. Building on the premise that absorptive capacity facilitates innovation (Müller et al., 2021) and drawing on self-efficacy theory, which emphasizes individuals’ beliefs in their capability to perform academic tasks (Bandura, 1977), this study argues that absorptive capacity does not automatically translate into academic success. Instead, its effects operate through students’ innovative attitudes, reflecting their willingness to engage with and apply newly acquired knowledge. Accordingly, we focus on perceived academic performance to capture students’ internal evaluations of competence. Empirically, the study focuses on Chinese business students enrolled in Korean universities, a rapidly growing yet underexplored population in international higher education, providing a timely context for examining learning processes in cross-border education.

This study makes three theoretical contributions to the education and educational psychology literature. First, it reconceptualizes absorptive capacity as an individual-level learning capability, extending the construct beyond organizational contexts to explain how students acquire, integrate, and apply knowledge in higher education. By shifting attention from outcome-based explanations to underlying learning mechanisms, the study advances process-oriented theorizing in student learning research. Second, the study identifies innovative attitudes as a key pathway through which absorptive capacity translates into academic engagement and performance, responding to calls for greater insight into how learning capacities are enacted in practice. Third, by framing academic performance as a perceived and internally evaluated outcome grounded in self-efficacy theory (Bandura, 1977), the study offers a theoretically coherent alternative to objective indicators that may overlook students’ subjective learning experiences. Together, these contributions clarify how learning capabilities and student attitudes jointly shape academic outcomes in international higher education settings.

## Literature review

Internationalization has become an increasingly prominent aspect of higher education globally (Levent, 2016), as a growing number of students pursue overseas education to advance their academic development (Kelo et al., 2006). International mobility not only facilitates the transfer of tacit knowledge embedded within individuals but also stimulates innovation of recipients through cross-border knowledge flows (Song et al., 2003; Rosenkopf and Almeida, 2003). Accordingly, international mobility functions as a mechanism of knowledge spillover that extends beyond the movement of financial and physical capital to include the exchange of intangible resources such as cultural understanding, linguistic competence, and diverse perspectives (Liu et al., 2015; Jeon, 2024). Nevertheless, while the macro-level benefits of mobility are well recognized, existing research has largely examined these processes through the lens of organizational absorptive capacity, offering limited insight into how individuals apply such capacity within international educational contexts (Knoben and Oerlemans, 2006; Cantner and Meder, 2007; Müller et al., 2021).

In educational research, absorptive capacity has likewise been examined primarily at the organizational level, with emphasis on institutional characteristics and learning environments such as organizational culture, leadership support, digital collaboration systems, teaching methods, and broader learning ecosystems (García-Morales et al., 2020; Peng et al., 2021; de Carvalho et al., 2022; Lenart-Gansiniec et al., 2022; Asiedu et al., 2023; Kim and Park, 2023). In contrast, individual-level absorptive capacity remains relatively underexplored, with only a limited number of studies addressing how students’ own learning capabilities shape educational outcomes (Peng et al., 2021; Fernández-Mesa et al., 2022). For example, Fernández-Mesa et al. (2022) report a positive association between students’ absorptive capacity and academic performance among management students in Spanish universities, while Peng et al. (2021) show that targeted instructional interventions can enhance absorptive capacity and improve learning outcomes among Chinese university students. Notably, these studies adopt an instructional design perspective, focusing on pedagogical inputs rather than the psychological processes through which individual absorptive capacity influences performance. Table 1 summarized prior research on absorptive capacity and highlights how the present study differs from existing approaches.

**Table 1:** Review of the literature on absorptive capity.

Authors	Independent variables	Dependent variables	Mechanisms	Moderators	Level of analysis	Context
Fabrizio (2009)	AC	performance	Knowledge processing	N/A	Organizational	Knowledge-intensive industry
Minbaeva et al. (2014)	High performance work practices	Knowledge effectiveness	Subsidiary AC	N/A	Organizational	Multinational corporations across international environments
Lowik et al. (2017)	Prior related knowledge, learning goal orientation, personality trait, network ties, organizational support	AC	Knowledge sharing behavior, individual creativity	N/A	Individual	Dutch high-tech firms
Li et al. (2020)	Specific pedagogical practice, Problem-based learning	Student employability	AC	Problem-based teaching	Individual	Higher education institutions
Lo and Tian (2020)	Knowledge sharing	Innovation capability	AC	N/A	Organizational	Higher education institutions
García-Morales et al. (2020)	Use of web 2.0 technologies	Students’ social entrepreneurial intentions	AC	N/A	Individual	Hier education institutions
Zhou et al. (2020)	HRM practice	Knowledge transfer effectiveness	AC	Cultural integration climate	Organizational	Cross-border M&A
Müller et al. (2021)	AC	Business model development	N/A	Innovation strategy	Organizational	Digital transformation
Peng et al. (2021)	Teaching strategy	Employability	AC	N/A	Individual	Educational sector
Fernández-Mesa et al. (2022)	AC	Academic success		Traditional and Innovative methodologies, Cooperative climate	Individual	Educational sector
Park et al. (2022)	Inward knowledge transfer, AC	Turnover intention of host-country nationals	Alignment between knowledge received and absorptive capacity	N/A	Individual	Host-country employees in an international business
Lenart-Gansiniec et al. (2022)	Prior knowledge, employees’ skills, educational projects, interactions with the environment	AC			Organizational	Higher education institutions
de Carvalho et al. (2022)	Perceived AC and Innovation	Organizational performance and Innovation		N/A	Organizational	Public higher education institution
Yao et al. (2022)	AC	Firm performance		Informal institutions	Organizational	International business
Asiedu et al. (2023)	AC	Innovation generation	Inter-functional Coordination	N/A	Organizational	Higher education institutions in a developing country
Kim and Park (2023)	Experiential learning	Entrepreneurial capability	AC	N/A	Individual	Higher education
Awwad et al. (2025)	AC	Financial performance	Innovation	N/A	Organizational	Business and finance
Cavallo et al. (2025)	External knowledge environment	AC	Internal communication efficiency	Quality of external knowledge, employees’ ability	Organizational	Open innovation environments
Chang et al. (2025)	Digital transformation anxiety	Digital innovation performance	Dynamic capability, AC	N/A	Organizational	Digital transformation
Lazzarotti et al. (2025)	Relationship depth with the university	Innovation process efficiency	N/A	Organizational routines for AC	Organizational	Industry-firm collaboration with the university
Current study	AC	Perceived academic performance	Innovative attitude	Motivation	Individual	Chinese students in Korean Universities

AC, Absorptive capacity.

To extend this line of inquiry, the present study examines individual-level absorptive capacity in international higher education, where students must interpret and apply knowledge under unfamiliar academic norms and institutional practices. Rather than treating learning outcomes as primarily determined by instructional conditions, we conceptualize international students as active knowledge processors whose academic success depends on their ability to recognize, integrate, and use new academic knowledge. This perspective draws on international management research, which highlights absorptive capacity as a key mechanism enabling effective knowledge application in cross-border contexts (Zhou et al., 2020; Park et al., 2022; Yao et al., 2022). Building on emerging educational research that extends absorptive capacity to the individual level (Li et al., 2020; Kim and Park, 2023), the present study focuses on how this capability becomes particularly consequential for students navigating international higher-education environments.

### Individual-level absorptive capacity and perceived academic performance

Absorptive capacity was originally conceptualized as an organization’s ability to recognize, assimilate, transform, and apply external knowledge (Cohen and Levinthal, 1990; Zahra and George, 2002). Importantly, absorptive capacity is not an outcome of learning, but a capability that enables knowledge use and learning. Recent research has extended this concept to the individual level, conceptualizing students’ absorptive capacity as their ability to acquire, integrate, and apply new academic knowledge in educational settings (Fernández-Mesa et al., 2022). This process-oriented view makes absorptive capacity a theoretically distinct antecedent to academic performance rather than a measure of learning achievement itself.

*Perceived academic performance* refers to students’ subjective assessment of their academic performance, shaped by their competence and personal goals (Kuncel et al., 2001; Izaguirre et al., 2023). Unlike objective academic performance, typically measured through quantifiable indicators such as GPA or standardized test scores (Credé and Kuncel, 2008), perceived academic performance is captured as an internal and psychological dimension of learning outcome. Grounded in Bandura’s (1977) self-efficacy theory, perceived academic performance reflects students’ beliefs in their capability to accomplish academic tasks and attain desired outcomes. Accordingly, this study focuses on perceived academic performance as an internalized evaluation of academic success, serving as a key psychological indicator of students’ satisfaction and overall learning experience.

Absorptive capacity functions as a critical individual capability that shapes students’ academic performance by facilitating effective engagement with new knowledge. Prior studies indicate that higher absorptive capacity is associated with improved learning-related outcomes, including employability and academic achievement (Peng et al., 2021; Fernández-Mesa et al., 2022; Kim and Park, 2023). For instance, Fernández-Mesa et al. (2022) found that students with stronger absorptive capacity achieved higher academic performance, as reflected in course grades.

Importantly, the relevance of absorptive capacity becomes especially pronounced in contexts where learners must navigate unfamiliar knowledge systems and heightened uncertainty. Research on international knowledge transfer suggests that absorptive capacity facilitates the internalization and application of external knowledge when individuals operate in novel institutional environments (Minbaeva et al., 2014; Zhou et al., 2020; Park et al., 2022; Yao et al., 2022). International students in host-country universities encounter such conditions as they engage with different pedagogical approaches and academic expectations. In this settings, students with higher absorptive capacity should be better positioned to integrate academic knowledge more effectively and evaluate their academic performance more positively. Accordingly, this study hypothesizes that:

H1: *The absorptive capacity of international students is positively related to their perceived academic performance.*

### Innovative attitude mediates the relationship between absorptive capacity and perceived academic performance

Why might students with higher absorptive capacity perceive themselves as performing better academically? This study proposes that an innovative attitude serves as a key psychological mechanism linking absorptive capacity to perceived academic performance. *Innovative attitude* reflects cognitive orientation toward engaging with new ideas, experimentation, and creative problem-solving (Ettlie and O’Keefe, 1982). In the educational context, an innovative attitude captures students’ propensity to approach learning actively, explore alternative strategies, and adapt flexibly to new academic demands. This distinction is critical to absorptive capacity, which represents what students can do, while an innovative attitude reflects how students choose to engage with learning opportunities.

Students with higher absorptive capacity are better equipped to process complex or unfamiliar knowledge, which reduces uncertainty when encountering new information. This capability fosters greater openness to experimentation and a willingness to adopt non-routine learning strategies, thereby cultivating an innovative attitude (Fernández-Mesa et al., 2022; Chao and Yu, 2022). In turn, such an attitude shapes how students interpret and evaluate their own academic experiences. Students who approach learning innovatively are more likely to perceive challenges as opportunities for growth, engage deeply with coursework, and attribute progress to their own adaptive efforts, processes that enhance self-assessments of academic performance.

Prior research supports this mechanism, such as Gong et al. (2009), who show that individuals’ aspirations to acquire new knowledge, an attitudinal orientation toward innovation, enhance creative outcomes, while Coronado et al. (2008) demonstrate that performance benefits emerge only when strong capabilities are accompanied by supportive innovation-oriented attitudes. Similarly, Lowik et al. (2017) find that individuals with high absorptive capacity are more likely to engage in innovation-related behaviors, and Kashan et al. (2025) show that absorptive capacity translates into innovation outcomes through knowledge conversion processes. Applied to education, these findings suggest that absorptive capacity alone does not directly translate into favorable performance perceptions unless it is activated through an innovation-related orientation.

Accordingly, we argue that an innovative attitude mediates the relationship between absorptive capacity and perceived academic performance, such that students with higher absorptive capacity develop stronger innovative attitudes, which in turn lead them to evaluate their academic performance more positively. Thus, we hypothesize:

H2: *Innovative attitude mediates the relationship between absorptive capacity and perceived academic performance.*

### The moderating role of motivation

An important factor that promotes students’ innovative attitude is their motivation. According to self-determination theory, motivation involves “energy, direction, persistence, and equifinality, all aspects of activation and intention” (Deci and Ryan, 2000, p. 69). In this study, motivation is not treated as an intervening process, as commonly addressed in prior research (Liu et al., 2024; Jaramillo-Mediavilla et al., 2024), but rather as a contingency factor that influences the strength of the relationship between absorptive capacity and innovative attitude. This perspective is theoretically grounded in the self-determination framework, which posits that individuals’ motivational intensity determines the extent to which their cognitive capabilities are activated and directed toward goal-directed action. While absorptive capacity represents students’ ability to acquire and process new knowledge, the extent to which this ability translates into innovation-oriented engagement depends on their motivational strength. Specifically, when students are highly motivated, they are more likely to transform their absorptive capacity into proactive and creative learning behavior, whereas students with low motivation may not fully leverage their absorptive capacity to achieve such outcomes.

Recent research supports this contingent view by demonstrating that the outcomes of knowledge-based capabilities depend critically on individuals’ motivational states. For example, contemporary work in educational and psychological contexts shows that absorptive capacity is more likely to translate into creative behavior when learners are actively engaged and driven to integrate external knowledge resources (Hassan et al., 2025). Likewise, studies on academic engagement suggest that motivation interacts with cognitive and learning-related capacities to enhance students’ persistence, participation, and performance, indicating that motivation strengthens the translation of these capacities into better academic outcomes (Reindl et al., 2022; Bureau et al., 2022). Motivation has also been identified as a central driver of adaptation and academic success, reinforcing the idea that capability effects are strongest when students possess the motivational energy to apply their knowledge in unfamiliar environments (Zhang et al., 2022; Liu et al., 2024). Taken together, these findings suggest that motivation intensifies the extent to which absorptive capacity fosters innovative attitudes, thereby strengthening its implications for academic performance.

H3: *Motivation moderates the relationship between absorptive capacity and innovative attitude, such that the relationship becomes stronger when motivation levels are high.*

H4: *Motivation moderates the relationship between absorptive capacity and perceived academic performance through innovative attitude, such that the relationship becomes stronger when motivation levels are high.*

**Figure 1:**
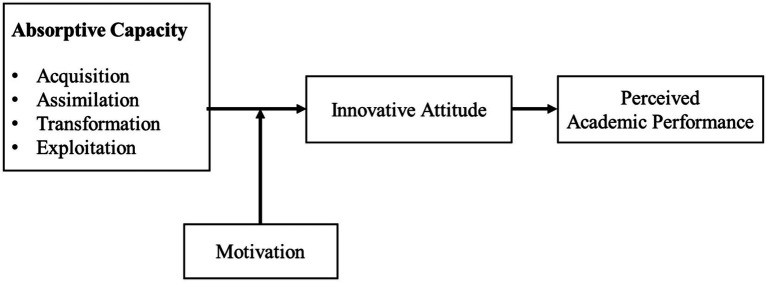
Research model.

The research model that we suggest is as follows (Figure 1). We propose that the absorptive capacity is positively related to perceived academic performance through innovative attitudes. Importantly, students’ motivation strengthens the positive effect of absorptive capacity on their perceived academic performance through innovative attitudes.

## Methodology

### Sampling

The purpose of this paper is to examine the relationship between Chinese students’ absorptive capacity and their perceived academic performance. Accordingly, the sample for this study comprised Chinese international students enrolled at universities in Seoul, South Korea. This population was selected because Chinese students currently represent the largest proportion of international students in Korea, making them a particularly relevant group for investigating cross-border learning dynamics.

Data were collected through a voluntary questionnaire administered to undergraduate and graduate students majoring in business (management) between April 2025 and July 2025. The questionnaire was originally developed in English and subsequently translated into Chinese following a rigorous forward-backwards translation procedure to ensure linguistic and cultural equivalence. First, two independent bilingual translators, both native in Chinese speakers, conducted separate forward translations from English into Chinese. Then, two different translators, native in English and blind to the original version, performed back-translations into English. The back-translation version was compared with the original to identify and correct any discrepancies.

After necessary adjustments were made to ensure clarity for Chinese students, the online survey link was distributed via email to 250 students. A total of 218 questionnaires were returned, yielding a response rate of 87%. After excluding incomplete responses and those that failed the attention check, 202 valid questionnaires remained. Among the respondents, 106 were female (52.5%), with an average age of 25 years (SD = 2.70). *A priori* power analysis using G∗Power indicated that a minimum sample size of 89 was required (effect size = 0.25, *α* = 0.05, and power = 0.90); thus, the final sample size of 202 met the statistical requirements.

### Measures

To measure absorptive capacity, this study employed a higher-order reflective construct (HOC) based on established literature (Zahra and George, 2002; Jansen et al., 2005; Flatten et al., 2011). The construct consisted of four reflective dimensions. The acquisition dimension (two items) assessed students’ ability to gather academic information and access external knowledge, the assimilation dimension (two items) measured their capacity to interpret and internalize new knowledge, the transformation dimension (three items) captured the extent to which students integrate new knowledge with existing understanding in response to external change, finally, the exploitation dimension (two items) evaluated students’ ability to apply prior knowledge to new academic tasks.

Students’ innovative attitude was assessed using the four-item scales (Scott and Bruce, 1994; De Jong and Den Hartog, 2010). Perceived academic performance was measured with the four-item scales (Pintrich and De Groot, 1990; Chemers et al., 2001; Marsh, 1993). By focusing on perceived rather than objective academic performance, this study conceptualized academic success as a psychologically internalized construct grounded in self-efficacy theory (Bandura, 1977). This approach aligns performance assessment with cognitive dimensions such as absorptive capacity and innovation-oriented attitude, while reducing potential inconsistencies arising from institutional differences in grading standards. Motivation was assessed using the three-item scales based on Vallerand et al. (1992) and Ryan and Deci (2000). Additionally, demographic variables were also collected and included as control variables in the analyses.

This study controlled for five factors that have been identified in prior research as potential influences on perceived academic performance: gender, age, length of study abroad, language difficulties, and GPA. Among these, language difficulties were measured using the five-item scales adapted from Yang and Clum (1995).

## Results

### Assessment of common method bias

Harman’s single-factor test was performed using principal component analysis on all items. The unrotated factor accounted for 36.21% of the total variance, which is below the 50% threshold for all observed indicators. Moreover, it is known that the one-way fit (χ^2^/df = 5.304, TLI = 0.701, CFI = 0.732, RMSEA = 0.146, SRMR = 0.092) was significantly worse than that of the four-factor model (χ^2^/df = 2.033, TLI = 0.928, CFI = 0.938, RMSEA = 0.072, SRMR = 0.053). Although these test is frequently employed as an initial check for common method bias, they may be insufficient for detecting subtle biases. To address this concern, we incorporated an unmeasured latent method factor into the confirmatory factor analysis model. The results showed nearly identical model fit after incorporating the latent method factor (χ^2^/df = 2.279, TLI = 0.898, CFI = 0.916, RMSEA = 0.080, SRMR = 0.070), and comparing the baseline model with a constrained model using the chi-square difference test was nonsignificant (*p* > 0.10), further indicating that common method bias was not an issue.

### Non-response bias

We divided the samples into two categories: early respondents (those in the first quartile) and late respondents (those in the third quartile), and explored the difference between the two groups (Singh and Kumar, 2010). The t-test comparison for these two groups with all four constructs (absorptive capacity, motivation, innovative attitudes, and perceived academic performance) indicated no significant differences between them, which validated that there was no non-response bias.

### Construct validity and reliability

The statistical analysis consisted of three steps using SmartPLS 4.0 software (Ringle et al., 2022). First, the measurement model was assessed using the partial least squares structural equation modeling (PLS-SEM). PLS-SEM is useful for analyzing complex models involving second-order constructs (Hair et al., 2012; Cuevas-Vargas et al., 2022). Second, the latent variables obtained from the PLS-SEM were examined using PLS-SEM bootstrapping with 5,000 subsamples to test the hypothesis. Lastly, a PLS prediction was conducted to evaluate the predictive ability of a PLS path model.

Table 2 shows the result of the analysis for the structure of the first-order and second-order constructs and their sub-variables. The factor loadings for all the items were substantial (>0.7), confirming the reliability of the measurement model (Hair et al., 2012). The variance inflation factor (VIF), an indicator of multicollinearity, was below 5, which is well under the empirical threshold of 10 (Kock, 2015); therefore, multicollinearity was not an issue. Cronbach’s alpha values and composite reliability (CR) exceeded 0.7, indicating a good level of reliability. Average variance extracted (AVE) was also examined to assess the convergent validity of the constructs, with all AVE values surpassing the recommended threshold of 0.5, thereby confirming convergent validity (Fornell and Larcker, 1981).

**Table 2:** Reflective measurement model assessment.

Constructs	Variable	Standard loading	VIF	Cronbach’s alpha	CR	AVE
Acquisition	Acqui1	0.906	1.955	0.783	0.783	0.822
	Acqui2	0.907	2.191			
Assimilation	Assi1	0.920	2.476	0.832	0.835	0.856
	Assi2	0.931	3.016			
Transformation	Trans1	0.879	2.710	0.843	0.844	0.761
	Trans2	0.877	2.277			
	Trans3	0.861	2.236			
Exploitation	Exploi1	0.932	2.766	0.843	0.844	0.865
	Exploi2	0.928	2.574			
Innovation attitude	InnoAtti1	0.849	2.066	0.906	0.907	0.780
	InnoAtti2	0.864	2.486			
	InnoAtti3	0.910	3.841			
	InnoAtti4	0.907	3.573			
Academic performance	AcPer1	0.843	2.217	0.877	0.878	0.731
	AcPer2	0.880	2.687			
	AcPer3	0.841	2.111			
	AcPer4	0.855	2.116			
Motivation	Motiv1	0.810	1.536	0.800	0.815	0.714
	Motiv2	0.885	1.913			
	Motiv3	0.837	1.837			

HOC	Latent variables	Path coefficient	Cronbach’s alpha	CR	AVE
Absorptive capacity	Acquisition	0.782	0.920	0.922	0.613
	Assimilation	0.884			
	Transformation	0.906			
	Exploitation	0.866			

VIF, variance inflation factor; CR, Composite Reliability; AVE, Average Variance Extracted.

Discriminant validity was assessed for the measurement model using the Fornell–Larcker criterion and the Heterotrait-Monotrait (HTMT) ratio. The result confirmed that the square root of all AVE values was higher than the estimated correlation between the components of this study (Fornell and Larcker, 1981), and all HTMT values were also lower than the threshold of 0.9 (Henseler et al., 2015) (Table 3), thus demonstrating acceptable discriminant validity. Additionally, model fit indices for the research model (SRMR = 0.055, d_ULS = 0.971, d_G = 0.497, Chi-square = 563.022, NFI = 0.816) fell within acceptable thresholds, confirming the model’s suitability for further analysis.

**Table 3:** Discriminant validity results.

Constructs	Acquisition AVE = 0.822	Assimilation AVE = 0.856	Transformation AVE = 0.761	Exploitation AVE = 0.865	Innovative attitude AVE = 0.780	Academic performance AVE = 0.731	Motivation AVE = 0.714
Fornell–Larcker criterion							
Acquisition	0.907						
Assimilation	0.614	0.925					
Transformation	0.566	0.753	0.872				
Exploitation	0.605	0.675	0.717	0.930			
Innovative attitude	0.460	0.478	0.494	0.498	0.883		
Academic performance	0.541	0.562	0.539	0.482	0.694	0.855	
Motivation	0.530	0.489	0.572	0.622	0.524	0.568	0.845
Heterotrait-monotrait ratio							
Acquisition							
Assimilation	0.761						
Transformation	0.696	0.896					
Exploitation	0.743	0.803	0.850				
Innovative attitude	0.544	0.549	0.561	0.566			
Academic performance	0.652	0.659	0.625	0.558	0.775		
Motivation	0.659	0.591	0.693	0.757	0.607	0.674	

### Model testing

**Figure 2:**
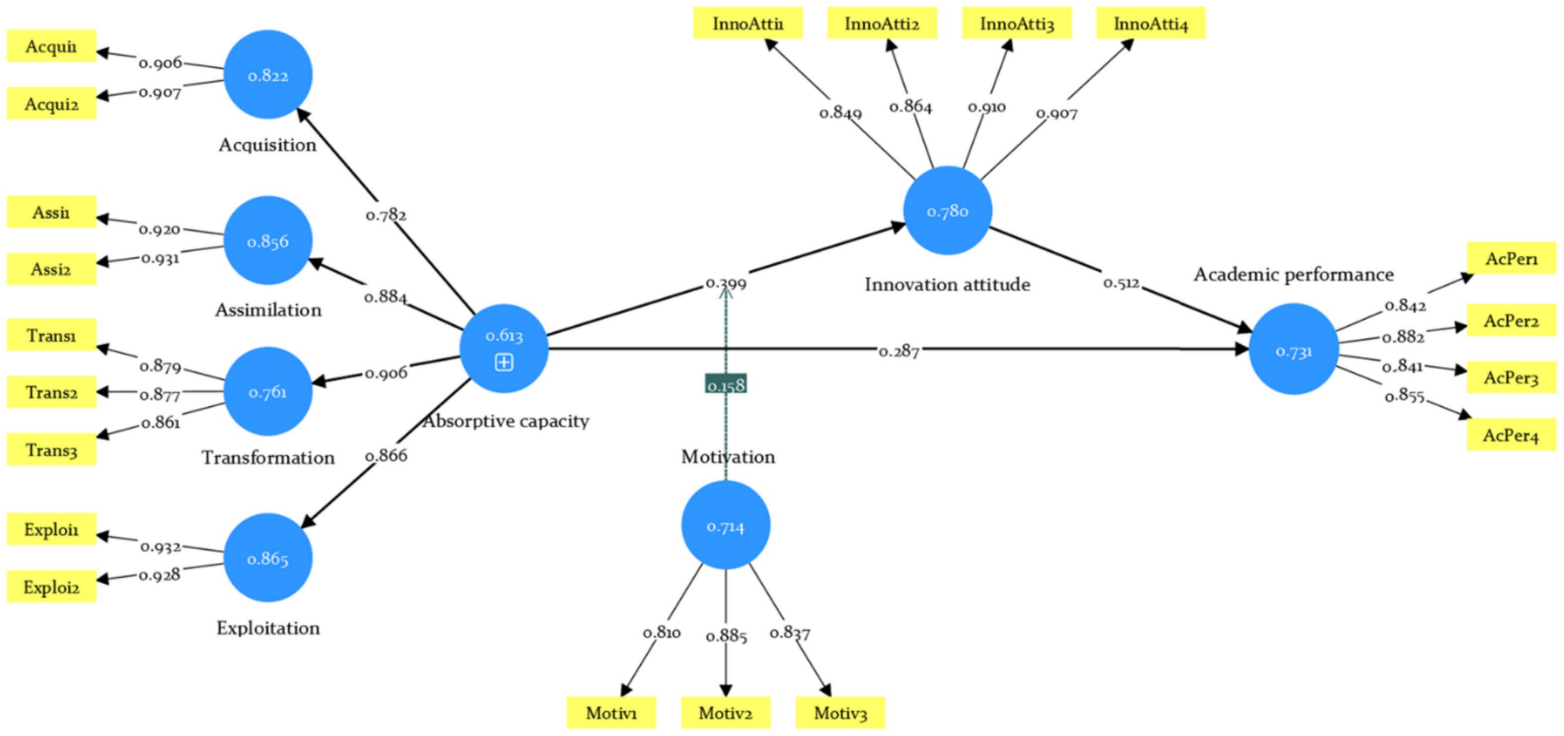
Structural model.

After controlling for gender, age, length of study abroad, language difficulties, and GPA, the path coefficient and its statistical significance were used to validate the hypothesis. The mediation analysis was conducted using bias-corrected bootstrapping with 5,000 resamples, and the results are presented in Figure 2 and Table 4.

**Table 4:** Hypothesis results.

Path	Standardized coefficient	Standard deviation (SD)	T values	*p* values	CI 5.0%	CI 95.0%
Control effects						
Gender → academic performance	0.026	0.093	0.282	0.778	−0.126	0.178
Age → academic performance	0.066	0.045	1.487	0.137	−0.005	0.141
Duration → academic performance	0.003	0.050	0.065	0.948	−0.080	0.086
GPA → academic performance	0.123	0.042	2.934	0.003	0.055	0.195
Languages → academic performance	−0.078	0.048	1.616	0.106	−0.159	0.000
Direct effects						
AC → innovative attitude	0.399	0.077	5.208	0.000	0.273	0.525
AC → academic performance	0.287	0.059	4.882	0.000	0.188	0.381
Innovative attitude → academic performance	0.512	0.058	8.813	0.000	0.409	0.601
Motivation → innovative attitude	0.304	0.074	4.112	0.000	0.181	0.423
Mediating effects						
Motivation → innovative attitude → academic performance	0.156	0.046	3.415	0.001	0.086	0.234
AC → innovative attitude → academic performance	0.204	0.041	4.971	0.000	0.143	0.278
Moderating effects						
AC x motivation → innovative attitude	0.158	0.068	2.318	0.021	0.024	0.241
+1 SD motivation	0.558	0.103	5.418	0.000	0.402	0.737
−1 SD motivation	0.241	0.102	2.352	0.019	0.089	0.410
Moderated mediating effects						
AC x motivation → innovative attitude → academic performance	0.081	0.036	2.267	0.023	0.015	0.128
+1 SD motivation	0.286	0.055	5.169	0.000	0.206	0.388
−1 SD motivation	0.123	0.054	2.296	0.022	0.047	0.216

AC, Absorptive capacity.

The absorptive capacity was positively related to perceived academic performance (*β* = 0.287, *p* < 0.001) and innovative attitude (*β* = 0.399, *p* < 0.001), thus H1 was supported. The mediating role of innovative attitude between absorptive capacity and perceived academic performance was significant (*β* = 0.204, *p* < 0.001), indicating that the effect of the absorptive capacity on the perceived academic performance was partially mediated through the innovative attitude. Thus, the innovative attitude plays a mediator role; H2 was supported.

**Figure 3:**
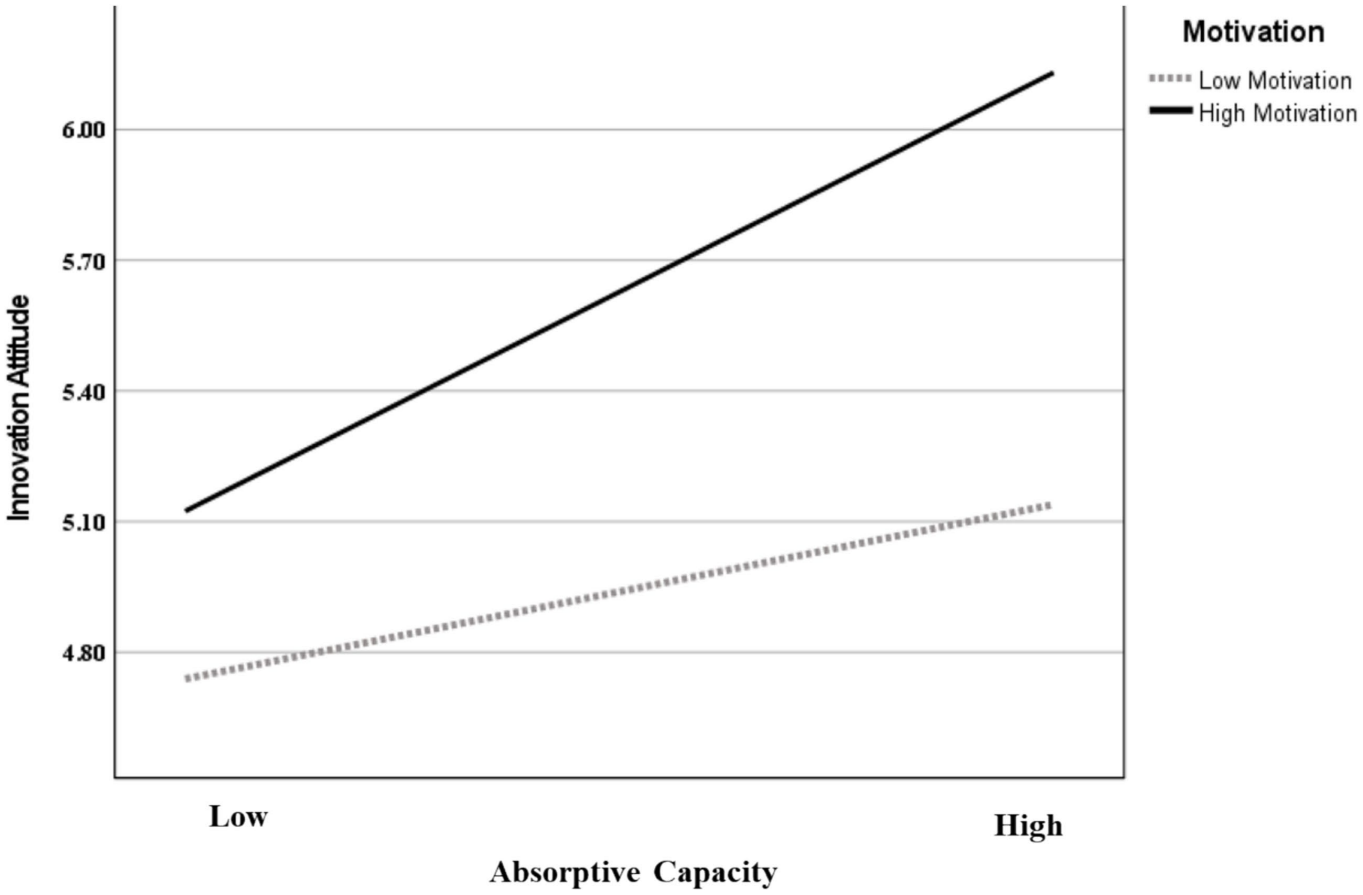
The interaction effect of absorptive capacity and motivation on innovative attitudes.

Moreover, the interaction effect of absorptive capacity and motivation (*β* = 0.158, *p* < 0.05) is positively related to innovative attitude, indicating that motivation strengthens the positive effect between absorptive capacity and innovative attitude. Thus, H3 was supported. To further verify this relationship, different levels of motivation (mean-centered), were explored to explore the influence of absorptive capacity on innovative attitude. As shown in Figure 3, when motivation is one standard deviation above the mean (+1 SD) (*β* = 0.558, *p* < 0.001), the slope of the positive relationship between absorptive capacity and innovative attitude is stronger, but this relationship is relatively weaker when motivation is one standard deviation below the mean (−1 SD) (*β* = 0.241, *p* < 0.05), thus H3 was supported (Table 4 and Figure 3).

The moderated mediation results reveal that the indirect impact of absorptive capacity and motivation on academic performance is mediated by the innovative attitude (*β* = 0.081, *p* < 0.05). Specifically, when motivation is low (−1 SD motivation), the conditional indirect effect is weaker (*β* = 0.123, *p* < 0.05); whereas when motivation is high (+1 SD motivation), the conditional indirect effect increases (*β* = 0.286, *p* < 0.001). These results signify that the mediation process through innovative attitude is more pronounced and impactful when motivation is high, highlighting the moderating influence in this pathway (Table 4). Accordingly, H4 was supported.

### Predictive relevance

The effect size (f^2^), coefficient of determination (R^2^), and predictive relevance (Q^2^) were examined. All values were within acceptable ranges, supporting the model’s predictive power (Cohen, 2013). Table 5 presents a summary of the predictive power results. The Q^2^ values for both perceived academic performance and innovative attitude were significantly greater than zero, indicating the model’s significance (Hair, 2014). Notably, the effect sizes range from small to medium, with the absorptive capacity’s direct effect on perceived academic performance representing a medium effect size. Although the mediation effect (H2) and the moderation effect (H3) are significant, their effect sizes are relatively small, suggesting a partial mediation effect.

**Table 5:** Effect size, coefficient of determination, and predictive relevance.

Constructs	Effect size(f^2^)		Coefficient of determination		Predictive relevance		
	Academic performance	Innovative attitude	R-square	R-square adjust	RMSE	MAE	Q-square
Academic performance			0.581	0.566	0.772	0.617	0.412
Innovative attitude	0.404		0.400	0.391	0.814	0.601	0.349
Motivation		0.090					
Absorptive capacity	0.128	0.156					
Absorptive capacity*motivation		0.068					

## Conclusion and discussion

This study advances understanding of academic success in international higher education by revealing how students’ absorptive capacity operates through psychological mechanisms to shape perceived academic performance. Focusing on Chinese students in Korean universities, the findings demonstrate that absorptive capacity is a central driver of students’ perceived academic performance. This result extends prior absorptive capacity research, which has largely focused on organizational outcomes (Müller et al., 2021; Yao et al., 2022; Kim and Park, 2023), by showing that absorptive capacity also functions as a micro-level capability enabling individuals to navigate unfamiliar academic environments. In this sense, international students emerge not as passive recipients of institutional knowledge but as active agents who process, integrate, and apply knowledge in cross-border learning contexts. Our results align with studies in international education and knowledge transfer contexts, which highlight that stronger absorptive capacity enhances adaptation and learning outcomes in cross-border settings (Minbaeva et al., 2014; Park et al., 2022; Zhou et al., 2020; Yao et al., 2022).

Importantly, the findings reveal that absorptive capacity influences perceived academic performance indirectly through students’ innovative attitude. This mediated relationship clarifies a key theoretical distinction between learning capability and learning orientation. While absorptive capacity reflects what students are cognitively capable of doing, innovative attitude captures how they choose to engage with learning opportunities, whether they approach tasks experimentally, seek alternative strategies, and view challenges as opportunities for growth. By empirically demonstrating this pathway, the study shows that cognitive capability alone is insufficient to shape performance perceptions unless it is translated into innovation-oriented engagement, consistent with prior findings linking learning capability to creative engagement (Fernández-Mesa et al., 2022; Lowik et al., 2017; Kashan et al., 2025). This process-based explanation refines existing learning frameworks by specifying how absorptive capacity is activated at the individual level.

Furthermore, the moderated mediation results indicate that motivation strengthens the translation of absorptive capacity into innovative attitude, consistent with self-determination theory (Deci and Ryan, 2000). Highly motivated students are more likely to convert their cognitive capabilities into exploratory and creative learning attitudes, whereas low motivation constrains this activation even when absorptive capacity is high. This finding highlights the contingent nature of learning processes in international education and underscores that capability, attitude, and motivation jointly shape academic outcomes, echoing patterns observed in diverse international educational settings (Chao and Yu, 2022; Zhang et al., 2022; Liu et al., 2024). Rather than treating these elements as independent predictors, the study demonstrates their interdependence, offering a more nuanced explanation of academic success.

Taken together, these findings provide a process-oriented account of learning in international higher education, in which academic success emerges from the interplay of cognitive capability (absorptive capacity), attitudinal orientation (innovative attitude), and motivational activation. By integrating these elements within a moderated mediation framework, the study offers a dynamic explanation of how and under what conditions international students perceive themselves as academically successful. This integrated perspective deepens theoretical understanding of learning and innovation at the individual level and highlights the importance of psychological mechanisms in cross-border educational settings.

### Practical implications

The findings of this study suggest that academic success among international students should be understood not merely as an outcome of ability or effort, but as a developmental process through which absorptive capacity fosters innovative attitudes and motivational engagement. Accordingly, universities and educators should design learning environments that strengthen students’ capacity to recognize, assimilate, and apply new academic knowledge, particularly in culturally unfamiliar contexts. Academic support programs such as structured orientation courses, reflective learning workshops, and peer-assisted study systems may enhance students’ absorptive capacity and help them adapt more effectively to new educational expectations.

In addition, perceived academic performance is not only an indicator of academic achievement but also a meaningful reflection of international students’ satisfaction and adaptation in Korean university contexts. Because international students often face unfamiliar academic norms and cultural expectations, how they internally evaluate their academic progress may shape their overall sense of belonging, confidence, and satisfaction with their university experience. Therefore, universities should monitor students’ perceived academic performance alongside objective indicators, as it may provide insight into students’ academic adjustment and well-being.

Importantly, higher perceived academic performance may also generate broader institutional benefits beyond individual learning outcomes. International students who feel academically successful are more likely to develop positive overall impressions of their host universities, which can strengthen student satisfaction, retention, and long-term engagement. Such positive experiences may further encourage favorable word-of-mouth communication within students’ home-country networks, enhancing the university’s international reputation and attractiveness to prospective students. In this sense, supporting students’ perceived success may function not only as an educational strategy but also as an important component of universities’ global recruitment and internationalization efforts.

### Limitation

Despite its contributions, this study has several limitations that provide important avenues for future research. First, the cross-sectional design restricts causal inference and prevents conclusions about the temporal ordering of absorptive capacity, innovative attitude, motivation, and academic performance. Since learning capabilities and motivational orientations may develop dynamically over time and may also exert reciprocal effects, future research should employ longitudinal or experimental designs to capture the evolving nature of these relationships in international learning contexts.

Second, although perceived academic performance is theoretically supported by self-efficacy perspectives and reflects students’ internal evaluation of competence, reliance on self-reported outcomes may introduce perceptual bias and cultural response tendencies. Future studies could integrate subjective assessments with objective indicators such as GPA, course grades, or behavioral engagement measures to strengthen construct validity and clarify the extent to which perceived success aligns with observable academic achievement.

Third, the study’s empirical focus on Chinese business students enrolled in Korean universities limits generalizability across cultural, disciplinary, and institutional settings. Cultural norms surrounding hierarchy, self-evaluation, and innovation may shape both absorptive capacity processes and attitude and motivational engagement differently across societies. Moreover, disciplinary contexts vary substantially in pedagogical expectations and performance standards, suggesting the need for comparative research across academic fields and student populations with different cultural distances and learning environments.

Finally, while this study highlights absorptive capacity, innovative attitude, and motivation as key explanatory mechanisms, international academic success is likely shaped by additional contextual and relational factors, including peer learning networks, instructional practices and cultural adjustment processes. Incorporating these variables would allow future research to develop a more integrative model of student learning and adaptation, thereby deepening the theoretical integration of absorptive capacity and self-determination frameworks in global higher education.

## Data Availability

The raw data supporting the conclusions of this article will be made available by the authors, without undue reservation.

## Appendix: all measurements used in this study

**Table:**

Variables		Items
Absorptive capacity (Zahra and George, 2002; Jansen et al., 2005; Flatten et al., 2011)	Acquisition1	I actively seek academic materials beyond those provided in class.
	Acquisition2	I actively interact with classmates or teachers to acquire new academic knowledge.
	Assimilation1	I can quickly understand new academic information related to my courses.
	Assimilation2	I can connect new knowledge with what I already know
	Transformation1	I can apply what I’ve learned to new academic tasks or solve new academic problems,
	Transformation2	I can combine knowledge from different subjects to deepen my understanding
	Transformation3	I adjust my understanding of a topic when I encounter new information.
	Exploitation1	I use academic knowledge when working on practical tasks such as group projects.
	Exploitation2	I modify my study habits according to what I have learned from previous experiences.
Innovative attitude (Scott and Bruce, 1994; De Jong and Den Hartog, 2010)	Innovation1	I like to actively seek opportunities to apply new logic and methods in my learning.
	Innovation2	I like to adapt and explore different approaches to tasks.
	Innovation3	I like to appreciate creative thinking and explore new innovation opportunities.
	Innovation4	I like to explore various problem-solving methods with curiosity.
Academic performance (Pintrich and De Groot, 1990; Chemers et al., 2001; Marsh, 1993)	Performanc1	I am satisfied with my overall academic performance.
	Performanc2	I achieve high scores in exams, assignments, or projects.
	Performanc3	I often receive positive academic feedback from my instructors.
	Performanc4	I believe my academic performance fully reflects my potential.
Motivation (Vallerand et al., 1992; Ryan and Deci, 2000)	Motivation1	I feel a sense of accomplishment when I do well academically.
	Motivation2	My motivation to complete assignments comes from wanting to achieve future success.
	Motivation3	Good grades motivate me to study hard.
